# Therapeutic approach to bronchiolitis: why pediatricians continue to overprescribe drugs?

**DOI:** 10.1186/1824-7288-36-67

**Published:** 2010-10-01

**Authors:** Daniele De Brasi, Fortunato Pannuti, Fabio Antonelli, Federica de Seta, Paolo Siani, Luciano de Seta

**Affiliations:** 1 Pediatric Unit, AORN "A. Cardarelli", Naples, Italy; 2 Pediatric Unit, San Paolo Hospital, Naples, Italy

## Abstract

**Background:**

Bronchiolitis guidelines suggest that neither bronchodilators nor corticosteroids, antiviral and antibacterial agents should be routinely used. Although recommendations, many clinicians persistently prescribe drugs for bronchiolitis.

**Aim of the study:**

To unravel main reasons of pediatricians in prescribing drugs to infants with bronchiolitis, and to possibly correlate therapeutic choices to the severity of clinical presentation. Also possible influence of socially deprived condition on therapeutic choices is analyzed.

**Methods:**

Patients admitted to Pediatric Division of 2 main Hospitals of Naples because of bronchiolitis in winter season 2008-2009 were prospectively analyzed. An RDAI (Respiratory Distress Assessment Instrument) score was assessed at different times from admission. Enrolment criteria were: age 1-12 months; 1^st ^lower respiratory infection with cough and rhinitis with/without fever, wheezing, crackles, tachypnea, use of accessory muscles, and/or nasal flaring, low oxygen saturation, cyanosis. Social deprivation status was assessed by evaluating school graduation level of the origin area of the patients. A specific questionnaire was submitted to clinicians to unravel reasons of their therapeutic behavior.

**Results:**

Eighty-four children were enrolled in the study. Mean age was 3.5 months. Forty-four per cent of patients presented with increased respiratory rate, 70.2% with chest retractions, and 7.1% with low SaO2. Mean starting RDAI score was 8. Lung consolidation was found in 3.5% on chest roentgenogram. Data analysis also unraveled that 64.2% matched clinical admission criteria. Social deprivation status analysis revealed that 72.6% of patients were from areas "at social risk". Evaluation of length of stay vs. social deprivation status evidenced no difference between "at social risk" and "not at social risk" patients. Following therapeutic interventions were prescribed: nasal suction (64.2%), oxygen administration (7.1%), antibiotics (50%), corticosteroids (85.7%), bronchodilators (91.6%). Statistically significant association was not found for any used drug with neither RDAI score nor social deprivation status. The reasons of hospital pediatricians to prescribe drugs were mainly the perception of clinical severity of the disease, the clinical findings at chest examination, and the detection of some improvement after drug administration.

**Conclusions:**

We strongly confirm the large use of drugs in bronchiolitis management by hospital pediatricians. Main reason of this wrong practice appears to be the fact that pediatricians recognize bronchiolitis as a severe condition, with consequent anxiety in curing so acutely ill children without drugs, and that sometimes they feel forced to prescribe drugs because of personal reassurance or parental pressure. We also found that social "at risk" condition represents a main reason for hospitalization, not correlated to clinical severity of the disease neither to drug prescription. Eventually, we suggest a "step-by-step" strategy to rich a more evidence based approach to bronchiolitis therapy, by adopting specific and shared resident guidelines.

## Introduction

Bronchiolitis is a virally-induced acute bronchiolar inflammation associated with signs and symptoms of airway obstruction. It is the most common lower respiratory tract infection in infants and represents a common reason for attendance in the Emergency Department (ED) and for hospital admission. Typical bronchiolitis in infants is a self-limited disease that is little modified by aggressive evaluations, use of antibiotics or other therapies. Guidelines on its management have been extensively produced to address diagnosis as well as various therapeutic interventions and prevention strategy. Cincinnati guidelines [1] suggest that neither bronchodilators nor steroids, antiviral and antibacterial agents should be routinely used. In particular, use of antibiotics and steroids should be strongly discouraged, whereas administration of bronchodilators or epinephrine may be considered as an option, particularly when there is a family history for allergy, asthma, or atopy. Moreover, recent evidences have demonstrated advantages of the use of inhaled hypertonic saline in improving clinical score and shortening duration of hospitalization [2,3]. On the other hand, it has been stated that clinical practice guidelines are intended to assist clinicians in decision-making, not replacing clinical judgment and not providing the only appropriate approach to the management of children with bronchiolitis [1].

Although guidelines recommendations, many clinicians persistently and routinely use drugs in bronchiolitis, including bronchodilators, steroids and antibiotics [4]. Many papers describe efforts to modify bronchiolitis therapeutic management, some reporting successful intervention toward international guidelines [5,6]. A similar effort in managing community acquired pneumonia (CAP) was recently produced by our group: a significant improvement on the management of patients with CAP has been recorded after the discussion and introduction of routine use of international guidelines [7].

Social risk represents a main reason for hospitalization and occasionally for more frequent therapeutic interventions, as reported for many diseases, including bronchiolitis [8,9]. Spencer et al. [10] reported that children living in more socially deprived areas appeared to be more than 1.5 times as likely to be admitted and to require a medical intervention (i.e. artificial ventilation, intravenous infusion, nasogastric feeding, oxygen treatment, or complications requiring treatment) than children living in other parts of the city. In a recent paper [11], we analyzed the influence of socially deprived condition on admission of children affected by CAP, and we found that social status represents the main reason for hospitalization, independently from clinical severity of the disease.

In the present study we analyze a group of infants admitted to Divisions of Pediatrics of 2 Southern Italy Hospitals with bronchiolitis, describe pediatricians therapeutic choices and try to evaluate main reasons for their therapeutic behavior.

## Methods

Patients with bronchiolitis admitted to Pediatric Divisions of AORN "A. Cardarelli" and "San Paolo" Hospital of Naples in winter season 2008-2009 were prospectively analyzed. Enrolment criteria were: 1-12 months aged infants with 1^st ^lower respiratory infection associated with at least one of the following: history of cough and rhinitis, wheezing, crackles, tachypnea, use of accessory muscles, and/or nasal flaring, low oxygen saturation (SaO2), cyanosis, with/without fever. Exclusion criteria were: cystic fibrosis, bronchopulmonary dysplasia (BPD), immune deficit disease, congenital heart failure (CHF). Clinical evaluation of patients was based on RDAI (Respiratory Distress Assessment Instrument) score assessed at 0, 12, 24 and 48 hours from admission, according to Langley et al. [12], with partial modification. In detail, modification concerns lung fields location of wheezing that was better defined, as illustrated in Table 1. In relation to severity, RDAI score <8 was attributed to mild and moderate forms, whereas > 9 to severe forms [13]. Admission criteria included clinical criteria (i.e. dehydration, high respiratory rate [RR, defined as high as > 60 breaths for minutes for 0-2 months babies, and > 50 breaths for minutes for 2-12 months], low SaO2 (< 92%), apnea, nasal flaring or grunting, severe chest wall retractions, cyanosis, poor feeding, lethargy, seizures, and mild to moderate symptoms in patients aged lower than 3 months) and non-clinical criteria, i.e. inability of family to care their child [1,14,15].

**Table 1:** RDAI (Respiratory Distress Assessment Instrument) score (from reference [6], modified).

		Score					
			
	Symptoms	0	1	2	3	4	Maximum score
	Expiration	None	End expiration	Half expiration	3/4^th ^expiration	Continuous	4
	
Wheezing	Inspiration	None	Partial	Continuous			2
	
	Location	None	< 2/4^th ^lung fields	> 3/4^th ^lung fields			2

	Supraclavicular	None	Mild	Moderate	Severe		3
	
Chest	Intercostal	None	Mild	Moderate	Severe		3
retractions							
	Subcostal	None	Mild	Moderate	Severe		3

	Total						17

Family ability to care children was assessed by evaluating the social deprivation status related to living areas of patients. It was indicated by school graduation area mapping of Naples city and surrounding areas, as previously described [11,16]. In detail, a "deprivation score" was generated based on education level, in respect to the geographic area of origin. Naples city and surrounding areas were analyzed. Prevalence of low school graduation people (evaluating primary school graduation, no school graduation literate, and illiterate people) of the single geographic area was considered the specific "area score" (data not shown). The higher was the score, the higher was the social risk. The "deprivation score" was generated as follows: single area score was compared with national (Italian) score (0.36), considered the "risk cut-off"; areas with a lower score (< 0.36) were considered "not at social risk"; areas with a higher score (> 0.37) were considered "at social risk".

Thirty clinicians from both Hospitals were aware on correct bronchiolitis guidelines for admission and therapeutic approach; however, neither specific training program on bronchiolitis management nor shared and discussed guidelines were previously diffused among them. The reasons of their therapeutic choices were analyzed by proposing them a specific questionnaire. For each used drug, namely antibiotics, steroids, and bronchodilators (albuterol), 7 questions were submitted (9 for antibiotics). Each question had multiple choices and more than one answer could be given for each question (see Table 2).

**Table 2:** Answers of pediatricians to the questionnaire on therapeutic choices in bronchiolitis: for each question, choices can be multiple and are not exclusive.

You prescribe antibiotics:	n. of answers
Because of clinical severity	33/76 (43%)

Only after chest roentgenogram or serologic test for bacterial infections	12/76 (15%)

To preserve from bacterial superinfections	8/76 (10%)

After 24-48 hours, if patient does not improve	7/76 (11%)

If patient is already on treatment	5/76 (6.5%)

Always (independently from clinical course)	5/76 (6.5%)

At beginning of the disease	3/76 (4%)

Because of detection of improvement after administration	3/76 (4%)

Just to do something and/or just for personal (medical) safety	0/76

*Total answers*	76

	

**You prescribe steroids:**	

Because of clinical severity	21/58 (36%)

On the base of chest clinical examination	12/58 (20%)

Because of detection of improvement after administration	9/58 (15.5%)

Always (independently from clinical course)	8/58 (14%)

If patient is already on treatment	2/58 (3%)

Just to do something and/or just for personal (medical) safety	2/58 (3%)

If patient does not improve	4/58 (7%)

*Total answers*	58

	

**You prescribe bronchodilators:**	

Because of detection of improvement after administration	21/57 (37%)

Because of clinical severity	13/57 (23%)

On the base of chest clinical examination	12/57 (21%)

Always (independently from clinical course)	6/57 (10%)

Never	3/57 (5%)

If patient is already in treatment	1/57 (3%)

Just to do something and/or just for personal (medical) safety	1/57 (2%)

*Total answers*	57

Statistic analysis was performed using the StatsDirect Statistical software version 2.7.5 (Stats Direct Ltd, Altrincham, UK). For comparison of effectiveness of different treatments/conditions, we used χ2 test based on 2 by k and r by c contingency table analysis and Fisher's exact test for smaller samples. Significance was defined as p < 0.05.

## Results

**Figure 1:**
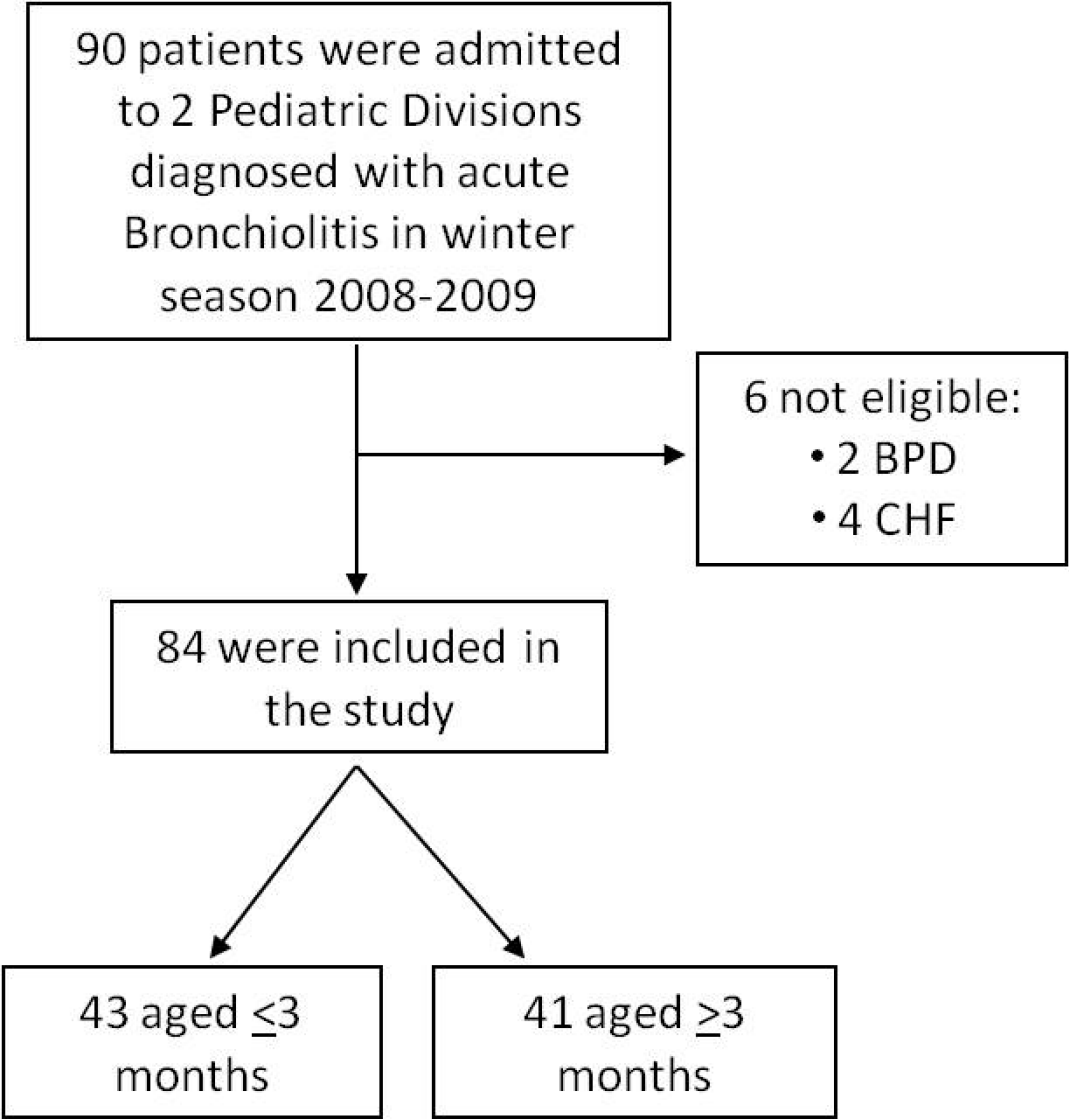
**Flow-chart describing patients enrolled and discharged from the study**. BPD, bronchopulmonary dysplasia; CHF, congenital heart failure.

Ninety infants were admitted to the 2 Hospitals because of bronchiolitis. Six were discharged because of BPD (2 patients) and CHF (4 patients) (Figure 1). Eighty-four children were enrolled in the study, 46 males (54%), 38 females (46%). Mean age was 3.5 months (range 1-12 months), 43 patients aged < 3 months (51.1%). Mean length of stay (LOS) was 4.8 days (range 1-10). Sixty-one patients directly accessed Emergency Department (E.D.)(72.6%), whereas 23 were sent by family pediatricians. Forty patients (47.6%) were already on treatment when admitted with either antibiotics, and/or corticosteroids, and/or bronchodilators. RR was measured in 63 patients out of 84, and 28/63 (44%) presented with increased RR, with mean starting RR of 51 (range 28-80). Fifty-nine (70.2%) children presented with chest retractions, and 6 (7.1%) with low SaO2 (lower than 92%). Twenty-two children out of 84 were pale at admission (26.1%) and 2/84 were cyanotic (2.3%). Seventy-seven patients had RDAI score assessment. Thirty-two out of 77 patients (41.5%) presented with a RDAI score > 9 at time 0, whereas 8 was mean starting RDAI. Mean RDAI score was 7.8 at 6 hours, 7.6 at 12 hours, 6.8 at 24 hours, and 6.9 at 48 hours. Chest roentgenogram was performed in 42 patients (50%), unraveling lung consolidation in 3 (3.5%)(Table 3).

**Table 3:** Clinical characteristics of admitted patients affected by bronchiolitis.

Clinical characteristics	n. of patients (%)	Clinical characteristics		n. of patients (%)
Total patients	84	Chest roentgenogram		42/84 (50%)

Males	46/84 (54%)	Lung consolidation		3/84 (3.5%)

Females	38/84 (46%)	Starting RDAI score of > 9		32/77 (41.5%)

Mean age (months)	3.5	RDAI (mean)	Time 0	8

1-3 months	43/84 (51.1%)		Time 6	7.8

4-12 months	41/84 (49%)		Time 12	7.6

Mean length of stay (LOS)	4.8 days		Time 24	6.8

Direct access to E.D.	61/84 (72.6%)		Time 48	6.9

Patients already on treatment	40/84 (47.6%)	Paleness		22/84 (26.1%)

Increased RR	28/63 (44%)	Cyanosis		2/84 (2.3%)

Patients with chest retractions	59/84 (70.2%)	Matching clinical admission criteria		54/84 (64.2%)
			
Patients with SaO2 < 92%	6/84 (7.1%)			

**Figure 2:**
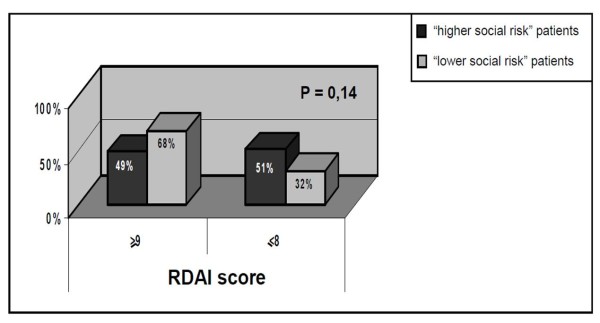
**RDAI score vs. social risk in admitted patients: among patients with a score ≥ 9, lower social risk is prevalent; among patients with a score ≤ 8, higher social risk is prevalent**. The difference is not statistically significant (P = 0.14).

Data analysis unraveled that 54/84 patients (64.2%) matched clinical admission criteria. Among 43 infants with bronchiolitis younger than 3 months, 18 presented with a mild form of the disease (33.3% of all clinically appropriated hospitalizations, 21.4% of total admitted patients): therefore, they were admitted mainly because of age < 3 months more than because of severe clinical impairment. On the other hand, social deprivation status analysis revealed that 61/84 admitted children (72.6%) were from living areas "at social risk"; among 30 patients not full-filling clinical criteria for hospitalization, 22 were from socially deprived areas (73.3%); among 18 infants aged < 3 months admitted with mild-moderate condition, 13 (72.2%) were from "at social risk" areas. In addition, analysis of clinical admission criteria vs. social deprivation status revealed that admitted patients with a higher social risk presented with RDAI scores equally distributed between mild and severe forms (i.e., among patients with high social risk, 29/57 [51%] with RDAI score < 8 vs. 28/57 [49%] with RDAI score > 9). On the other hand, among inpatients with lower social risk, higher RDAI scores were prevalent (13/19 [68.5%] with RDAI score > 9 vs. 6/19 [31.5%] with RDAI score < 8). Nevertheless, statistical analysis revealed that the difference was not significant (P = 0.14)(Figure 2).

Evaluation of LOS vs. social deprivation status evidenced no difference between "at social risk" and "not at social risk" patients in mean LOS (4.836 days, and 4.625 days, respectively) and in the number of days of staying at hospital (less or more than 5 days)(P = 0.67)(Additional file 1).

Therapeutic interventions on admitted infants with bronchiolitis were as follows: nasal suction in 54 (64.2%), oxygen administration in 6 patients (7.1%), antibiotics in 42 (50%), corticosteroids in 72 (85.7%), bronchodilators in 77 (91.6%). Seven patients (8.3%) required i.v. fluids administration. Combination therapy using together antibiotics, steroids and bronchodilators was prescribed in 34 patients (40.4%), bronchodilators and steroids in 32 patients (38.0%), whereas antibiotics associated with corticosteroids and with bronchodilators, in 6 (7.1%) and 1 patients (1.2%), respectively. Use of antibiotics, steroids or bronchodilators alone was scored in 2 (2.3%), 5 (5.9%), and 4 (4.7%) patients, respectively (Table 4). Eventually, all admitted patients received drug treatment during hospitalization (i.e., antibiotics and/or steroids and/or bronchodilators). Analysis of drugs in dependence on clinical severity unraveled no correlation among antibiotics, bronchodilators, or steroids and RDAI score. In particular, antibiotics were used in 20 mild cases and in 20 severe cases; conversely, they were not used in 25 severe cases and in 19 mild cases, respectively. Bronchodilators were used in 31 mild cases and in 37 severe cases, whereas in 7 mild and 9 severe cases they were not used. Last, corticosteroids were used in 39 mild cases and in 31 severe cases; on the contrary, they were not used in 3 mild and in 4 severe cases, respectively. The differences were not statistically significant (P = 0.53 for antibiotics, P = 0.89 for bronchodilators, P = 0.39, for corticosteroids)(Additional file 2). We found same result if the use of a single drug was related to social deprivation status. In particular, antibiotics were used in 42 patients: 33 were socially deprived, 9 were not; among 42 patients not treated with antibiotics, 28 were "at social risk", and 14 were not (P = 0.17). As to bronchodilators, they were used in 77 patients, 55 socially deprived and 22 not socially deprived; 7 patients were not treated with bronchodilators, 6 "at social risk", and a single patient "not at social risk" (P = 0.37). Last, we prescribed steroids to 72 patients: 50 were socially deprived, 22 were not; among 12 patients not treated with steroids, 11 were "at social risk", and a single patient was not (P = 0.07)(Additional file 3).

**Table 4:** Therapeutic interventions of admitted patients affected by bronchiolitis.

Interventions	n. of patients (%)
Nasal suction	54/84 (64.2%)

Oxygen administration	6/84 (7.1%)

I.v. fluids	7/84 (8.3%)

Antibiotics (total)	42/84 (50%)

Steroids (total)	72/84 (85.7%)

Bronchodilators (total)	77/84 (91.6%)

Antibiotics + Steroids	6/84 (7.1%)

Steroids + Bronchodilators	32/84 (38.0%)

Antibiotics + Bronchodilators	1/84 (1.2%)

Antibiotics + Steroids + Bronchodilators	34/84 (40.4%)

Antibiotics (alone)	2/84 (2.3%)

Steroids (alone)	5/84 (5.9%)

Bronchodilators (alone)	4/84 (4.7%)

Forty out of 84 patients (47.6%) were already on treatment when admitted, 11 with antibiotics, steroids and bronchodilators, 12 with antibiotics and steroids, 6 with steroids and bronchodilators, 2 with antibiotics and bronchodilators, 7 with steroids alone, 1 with antibiotics alone, 1 with bronchodilators alone. Forty-four patients out of 84 (68.1%) were out of treatment at home: all they started treatment in hospital, 22 showing a RDAI score > 9, and 22 a RDAI score < 8, at any time during hospitalization.

Reasons of pediatricians' therapeutic choices were detected by a specific questionnaire. Thirty pediatricians from 2 Hospitals were involved. They provided 76 answers about antibiotics use, 58 about steroids, and 57 about bronchodilators. Answers showed that antibiotics were usually prescribed because of the perception of clinical severity of the disease (43%), frequently at beginning of hospitalization (18%), and in some cases to preserve from possible bacterial superinfections (10%), or after 24-48 hours if patient did not improve (9%). As to steroids use (either inhaled or systemic), they were usually prescribed in dependence of clinical severity (36%) or depending on chest clinical examination (20%); a marginal group always prescribes steroids, independently from clinical course (14%) or because they detect some improvement after their administration (15%). Last, bronchodilators were used because pediatricians detected some improvement after their administration (37%) and in dependence of severe clinical course (23%) or because of chest clinical examination (21%). Interestingly, therapeutics prescription "just to do something and/or just for personal (medical) safety" was also indicated in a minority of cases (3% for steroids, 1.5% for bronchodilators)(for detail, see Table 2).

## Discussion

We studied 84 patients affected by bronchiolitis admitted to Divisions of Pediatrics of 2 Southern Italy main Hospitals to analyze characteristics of admitted patients, pediatricians' therapeutic choices and the reasons of their choices.

Most of admitted patients matched admission criteria (64.2%). However, analyzing data in detail, it appears that most of them were not severely affected, as only a very small group presented with SaO2 < 92% (6%), 28.5% of children presented with paleness or cyanosis, and mean starting RDAI score was 8 (indicating a moderate condition). Moreover, 21.4% of patients, although a mild form of bronchiolitis, were admitted because of age less than 3 months.

Data analysis also revealed that 72.6% of admitted children were from living areas "at social risk", most of them not full-filling clinical criteria for hospitalization: it is probable that those children were admitted because of supposed inability of their family to cure them at home, more than because of severe impairment. Evaluation of socioeconomic factors in determining admission and therapeutic choices for patients accessing E.D. has been already carried out for many diseases, demonstrating a direct correlation [17,18]. Same findings were reported for bronchiolitis. Jannson et al. [19] evaluated the influence of socioeconomic factors on the hospitalization of infants with bronchiolitis. Hospitalization rates were determined for the infants living in the 10 different administrative residential areas of Malmö (SWE) and correlated with socioeconomic factors in the respective residential areas. The severity of the disease was assessed by comparing oxygen saturation, days with supplemental oxygen and length of stay. Hospitalization rates varied more than fourfold between the 10 residential areas: infants living in the areas with the highest social burden were hospitalized almost twice as often as those from the rest of the city, but the severity of the disease was similar. Authors concluded that socioeconomic factors may have a significant influence on the hospitalization rate in bronchiolitis. In 1996, Spenser et al. [10] designed a case-control study of 307 infants admitted to Sheffield Hospitals (UK) with clinically suspected bronchiolitis, subsequently ascertained from laboratory records of nasopharyngeal aspirates cultured for respiratory syncytial virus. Social deprivation status was established by evaluating postcodes converted to electoral wards which were assigned Townsend deprivation index scores [20]. They demonstrated that in infants admitted with clinically suspected bronchiolitis socioeconomic deprivation is associated with an increased risk of admission and requirement of medical interventions. They also found this correlation even after taking into account parental smoking, and if only more severe cases were considered. Link between social deprivation and hospitalization/medical interventions was related as much to increased risk of significant morbidity as to professional behavior. These data partially overlap our observation: actually, from our data it seems that deprived infants presented with clinical signs and symptoms less severe than "not at risk" patients, and did not receive more therapeutic interventions than "not at risk" infants, even if this observation is not statistically significant (see Figure 1). On the other hand, the analysis of Spenser et al. [10] demonstrated that more deprived patients require more medical interventions because of a more severe condition, even if in their analysis Spenser et al. [10] did not accurately correlate clinical severity of signs and symptoms with deprivation status, as performed in the present study. In conclusion, a socially deprived condition does not appear to be correlated to a more severe form of bronchiolitis and does not represent a significant reason for drugs' prescription in patients admitted to our Hospitals.

Analysis of mean LOS in hospital did not evidence difference between "at social risk" and "not at social risk" population. Even if it would be expected that socially deprived patients spend a shorter time in hospital because of a less severe form of bronchiolitis, it is reasonable to consider that they usually stay longer in hospital because of family difficult to cure their children at home.

Treatment of bronchiolitis is controversial. Recently, literature extensively demonstrated the ineffectiveness of most of drugs. Apart from nasal suction [1], inhaled hypertonic saline [2,3], oxygen and i.v. fluids administration when necessary [1], and epinephrine in particular circumstances (especially in combination with oral steroids) [21], other interventions are not appropriate in respect to international guidelines. On the other hand, it is stated that clinical practice guidelines are intended to assist clinicians in decision-making, not replacing clinical judgment in diagnostic and therapeutic choices. The fact is that pediatricians still largely use drugs in bronchiolitis management, even if aware of this inappropriate practice [4]. Many reports on use of drugs in bronchiolitis can be found in the literature, particularly on steroids and antibiotics [22-24]. In a Cochrane review, it is reported that antibiotics are used at rates of 34 to 99% in uncomplicated cases of bronchiolitis [4]. In a study of the Dutch Pediatric Respiratory Society [25] a questionnaire on the use of diagnostic and therapeutic procedures and prescription of drugs after discharge was mailed to 110 hospital-based pediatric practices. A great deal of variation in management of bronchiolitis was found between respondents. Most used supplemental oxygen (100%) and tube feeding (96%) when needed, and gave nebulized bronchodilators, either as a trial (59%) or in a fixed schedule (33%). Antibiotics for suspected bacterial co-infection were used in 69% of cases. Corticosteroids were used for severe cases by 35% of respondents, whereas ribavirin in 11% of hospitals for treatment of children from high-risk groups. They concluded that a considerable variation in management of bronchiolitis exists between hospitals in the Netherlands, and several therapeutic approaches are used which are not evidence based, probably reflecting the lack of therapeutic options with proven clinical efficacy for this condition [25]. In the present study, we found that antibiotics were used in 50% of cases, corticosteroids in 85%, and bronchodilators in 91%, and that at least one of these drugs was used in all hospitalized patients. Even if a quiet correspondence in antibiotics use between the Dutch study [25] and our data was found (69% vs. 50%), both bronchodilators and steroids were more frequently used by our group than the Dutch one (59% in the Dutch study vs. 91% in our study for bronchodilators, and 35% in the Dutch study vs. 85% in present study for steroids). Furthermore, we found that drugs were not used in dependence to clinical severity and socially deprived condition, as demonstrated by the absence of correlation among drugs' use and RDAI score or social risk. However, a more accurate data examination reveals that a slight difference was found in use of steroids between social deprived and not deprived patients: it seems that steroids were prescribed more frequently for not deprived infants, even if this difference was not fully statistically significant (P = 0.07). If this difference would be true, a possible explanation could be that infants with a better social condition presented a more severe form of bronchiolitis, even if the difference with socially deprived patients appeared not statistically significant (see Figure 2). Nevertheless, a possible bias due to the small size of the sample of patients not treated with steroids (12 infants) should be taken into account.

Almost half of patients (47.6%) were already on treatment when admitted to our Divisions and therapy was generally confirmed in hospital. Even if this could represent a possible reason for hospital pediatricians to continue therapy on admission, it can not explain why the latter half of patients without home treatment started drugs after hospitalization, not in dependence of clinical severity. A possible explanation of this behavior is that bronchiolitis often appears to clinicians as a severe condition (particularly in hospitalized patients) always requiring aggressive therapeutic intervention. Also parents and other family members often think to bronchiolitis as a severe disease, seeing their baby to breath with difficulty, to suck or eat weakly, sometimes presenting with an exhausting cough: frequently their anxiety influences therapeutic choices of clinicians.

We also asked to pediatricians the reasons for their drug choices in bronchiolitis by inviting them to fill in a specific questionnaire. Examining their answers it appears that drugs were prescribed primarily because of the perception of clinical severity (43% of answers for antibiotics, 36% for steroids, 22% for bronchodilators), or on the basis of chest clinical examination (21% for bronchodilators, 20% for steroids), and also because of some improvement after their administration (37% for bronchodilators, 15% for steroids). Interestingly, also therapeutics prescription "just to do something and/or just for personal (medical) safety" was also indicated in a minority of cases (3% for steroids, 1.5% for bronchodilators). This behavior points out that bronchiolitis is thought as a serious condition by pediatricians, and that medical operators sometimes feel "forced" to do something in managing these children, because of personal safety or parental pressure.

## Conclusions

Aim of the present study was to clarify the reasons of a known wrong behavior by pediatricians in curing bronchiolitis. No correlation between clinical severity of the disease and drug prescription has been found. Also the analysis of drug prescription related to social deprivation status revealed absence of statistic significant difference. Moreover, the study unravels that bronchiolitis is perceived as a severe condition by pediatricians, "forced" to do something by parents or because of personal safety.

To override this wrong behavior, we will try to extensively diffuse specific and fully shared resident guidelines among hospital (and primary) pediatricians to rich an evidence based approach to bronchiolitis therapy using a "step-by-step" strategy, as demonstrated by previous successful experiences by our [7] and other groups [6].

## Competing interests

The authors declare that they have no competing interests.

## Authors' contributions

DDB and FdS conceived the study and drafted the manuscript. FA and FP collected data and analyzed results. LdS and PS conceived the study and polished the manuscript.

All authors read and approved the final manuscript.

## Supplementary Material

Additional file 1 **Length of stay vs. social risk**. Staying < 5 days and > 5 days were compared between "at social risk "and "not at social risk" patients: no statistical difference was evident between the two groups (P = 0.67). Click here for file

Additional file 2 **Use of drugs in dependence to clinical severity score (RDAI)**. No significant association is evident among RDAI score and either antibiotics (A), or bronchodilators (B), or steroids (C). Severe forms are considered forms with RDAI score > 9; mild forms are considered forms with RDAI score < 8. ATB, antibiotics; B2, bronchodilators; CS, steroids. Click here for file

Additional file 3 **Drugs vs. social risk**. No statistically significant difference is evident between social risk and use of antibiotics (A), bronchodilators (B), steroids (C). SR: social risk. + = presence, - = absence. Click here for file

## References

[B1] Bronchiolitis Guideline Team, Cincinnati Children's Hospital Medical Center Evidence based clinical practice guideline for medical management of bronchiolitis in infants 1 year of age or less presenting with a first time episode 2005 1 1 13 http://www.cincinnatichildrens.org/svc/alpha/h/health-policy/ev-based/bronchiolitis.htm

[B2] Wainwright C Acute viral bronchiolitis in children- a very common condition with few therapeutic options Paediatr Respir Rev 2010 11 39 45 10.1016/j.prrv.2009.10.001 20113991 PMC7106315

[B3] Zorc JJ Hall CB Bronchiolitis: recent evidence on diagnosis and management Pediatrics 2010 125 342 349 10.1542/peds.2009-2092 20100768

[B4] Spurling GKP Fonseka K Doust J Del Mar C Antibiotics for bronchiolitis in children Cochrane Database of Systematic Reviews 2007 1 CD005189 10.1002/14651858.CD005189.pub2 17253545

[B5] Barben J Kuehni CE Trachsel D Hammer J Swiss Paediatric Respiratory Research Group Management of acute bronchiolitis: can evidence based guidelines alter clinical practice? Thorax 2008 63 1103 1109 10.1136/thx.2007.094706 18723582

[B6] Touzet S Réfabert L Letrilliart L Ortolan B Colin C Impact of consensus development conference guidelines on primary care of bronchiolitis: are national guidelines being followed? J Eval Clin Pract 2007 13 651 656 10.1111/j.1365-2753.2007.00781.x 17683310

[B7] Siani P de Seta L Saitta F Antonelli F Niglio B Pannuti F De Vivo M Cioffi L Causa P Metafora P Ercolini P Miglietta A Montini T Rasca G Esposito A Le polmoniti di comunità: migliorarne il trattamento sia sul territorio che in ospedale con un protocollo comune Medico e Bambino pagine elettroniche 2008 10 http://www.medicoebambino.com/?id=RI0810_20.html

[B8] O'Donnell DR Parslow RC Draper ES Deprivation, ethnicity and prematurity in infant respiratory failure in PICU in the UK Acta Paediatr 2010 99 1186 1191 10.1111/j.1651-2227.2010.01803.x 20236254

[B9] Jansson L Nilsson P Olsson M Socioeconomic environmental factors and hospitalization for acute bronchiolitis during infancy Acta Paediatr 2002 91 335 338 10.1080/08035250252834021 12022309

[B10] Spencer N Logan S Scholey S Gentle S Deprivation and bronchiolitis Arch Dis Child 1996 74 50 52 10.1136/adc.74.1.50 8660048 PMC1511601

[B11] Antonelli F De Brasi D Siani P Appropriateness of hospitalization for CAP-affected pediatric patients: report from a Southern Italy General Hospital Ital J Pediatr 2009 35 26 10.1186/1824-7288-35-26 19725971 PMC2753332

[B12] Langley JM Smith MB LeBlanc JC Joudrey H Ojah CR Pianosi P Racemic epinephrine compared to salbutamol in hospitalized young children with bronchiolitis; a randomized controlled clinical trial BMC Pediatr 2005 5 7 10.1186/1471-2431-5-7 15876347 PMC1142326

[B13] Gupta P Aggarwal A Gupta P Sharma KK Oral salbutamol for symptomatic relief in mild bronchiolitis a double blind randomized placebo controlled trial Indian Pediatr 2008 45 547 553 18695272

[B14] Lind I Gill JH Calabretta N Polizzotto M What are hospital admission criteria for infants with bronchiolitis? J fam pract 2006 55 67 69 16388772

[B15] Walsh P Rothenberg SJ O'Doherty S Hoey H Healy R A validated clinical model to predict the need for admission and length of stay in children with acute bronchiolitis Eur J Emerg Med 2004 11 265 272 10.1097/00063110-200410000-00005 15359199

[B16] Krieger N Overcoming the absence of socioeconomic data in medical records: validation and application of a census-based methodology Am J Public Health 1992 82 703 710 10.2105/AJPH.82.5.703 1566949 PMC1694121

[B17] Barnett R Lauer G Urban deprivation and public hospital admissions in Christchurch, New Zealand, 1990-1997 Health Soc Care Community 2003 11 299 313 10.1046/j.1365-2524.2003.00425.x 14629201

[B18] Glazier RH Badley EM Gilbert JE Rothman L The nature of increased hospital use in poor neighborhoods': findings from a Canadian inner city Can J Public Health 2000 91 268 273 10986783 10.1007/BF03404286 PMC6979985

[B19] Jansson L Nilsson P Olsson M Socioeconomic environmental factors and hospitalization for acute bronchiolitis during infancy Acta Paediatr 2002 91 335 338 10.1080/08035250252834021 12022309

[B20] Townsend P Phillimore P Beattie A Inequalities in health in the Northern region 1986 Newcastle upon Tyne: Northern Regional Health Authority and Utniversity of Bristol

[B21] Johnson DW Patel H Wiebe N Correll R Brant R Mitton C Gouin S Bhatt M Joubert G Black KJ Turner T Whitehouse S Klassen TP Pediatric Emergency Research Canada (PERC) Epinephrine and dexamethasone in children with bronchiolitis N Engl J Med 2009 360 2079 2089 10.1056/NEJMoa0900544 19439742

[B22] Christakis DA Cowan CA Garrison MM Molteni R Marcuse E Zerr DM Variation in inpatient diagnostic testing and management of bronchiolitis Pediatrics 2005 115 878 884 10.1542/peds.2004-1299 15805359

[B23] Barben J Kuehni CE Trachsel D Hammer J Management of acute bronchiolitis: can evidence based guidelines alter clinical practice? Thorax 2008 63 1103 1109 10.1136/thx.2007.094706 18723582

[B24] Subcommittee on diagnosis and management of bronchiolitis Diagnosis and management of bronchiolitis Pediatrics 2006 118 1774 1792 10.1542/peds.2006-2223 17015575

[B25] Brand PL Vaessen-Verberne AA Differences in management of bronchiolitis between hospitals in The Netherlands. Dutch Paediatric Respiratory Society Eur J Pediatr 2000 159 343 7 10.1007/s004310051284 10834519

