# Supplementary material for: Therapeutic approach to bronchiolitis: why pediatricians continue to overprescribe drugs?
Source: Ital J Pediatr. 2010 Oct 1;36:67. doi: 10.1186/1824-7288-36-67 (PMC2958958; doi:10.1186/1824-7288-36-67)
Supplement: Additional file 1 — Length of stay vs. social risk. Staying < 5 days and > 5 days were compared between "at social risk "and "not at social risk" patients: no statistical difference was evident between the two groups (P = 0.67). [file 1824-7288-36-67-S1.PDF]

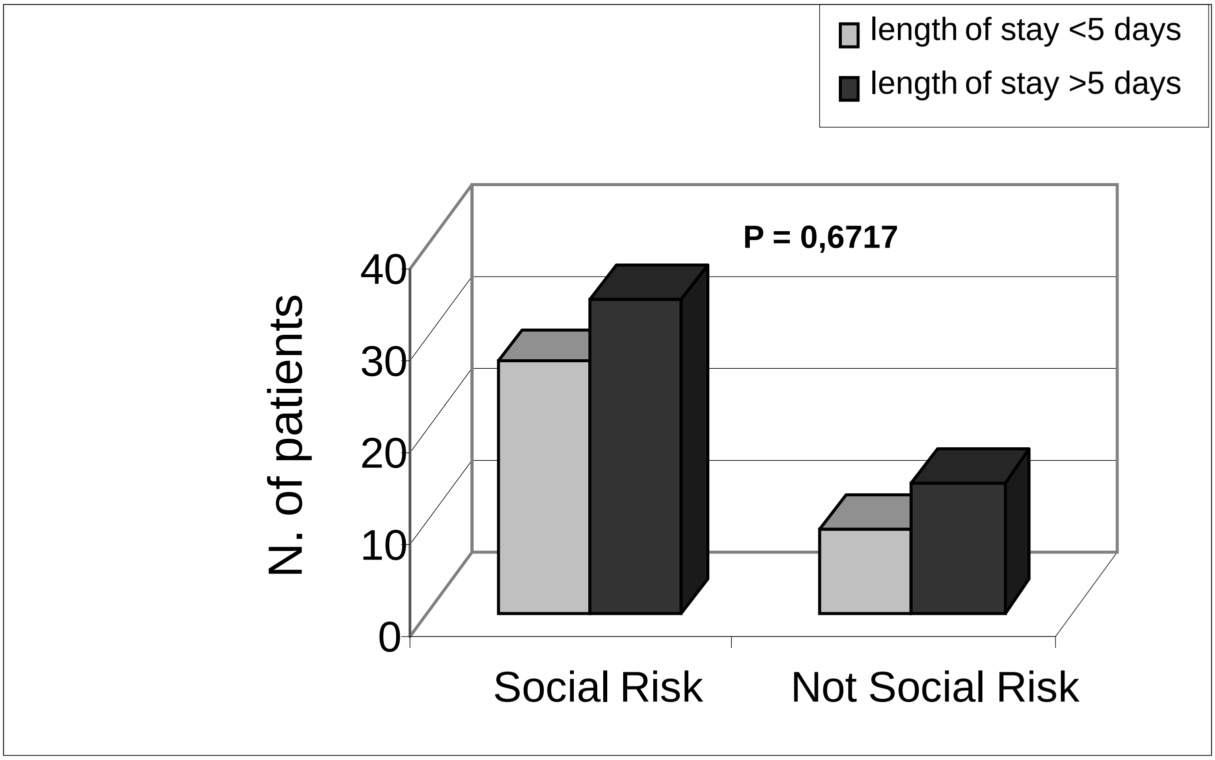

Length of stay vs. social risk.
Staying <5 days and >5days were compared between “at social risk “and “not at social risk“ patients: no statistical difference was evident between the two groups. (P = 0.67)
