# Supplementary material for: Therapeutic approach to bronchiolitis: why pediatricians continue to overprescribe drugs?
Source: Ital J Pediatr. 2010 Oct 1;36:67. doi: 10.1186/1824-7288-36-67 (PMC2958958; doi:10.1186/1824-7288-36-67)
Supplement: Additional file 2 — Use of drugs in dependence to clinical severity score (RDAI). No significant association is evident among RDAI score and either antibiotics (A), or bronchodilators (B), or steroids (C). Severe forms are considered forms with RDAI score > 9; mild forms are considered forms with RDAI score < 8. ATB, antibiotics; B2, bronchodilators; CS, steroids. [file 1824-7288-36-67-S2.PDF]

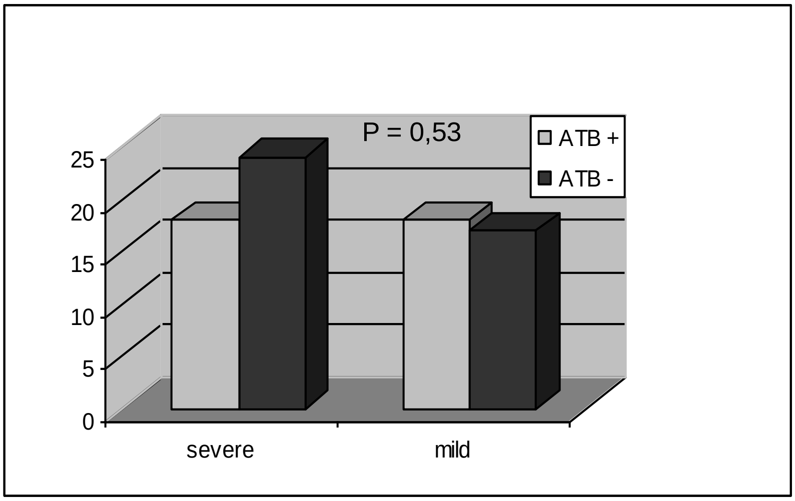

A

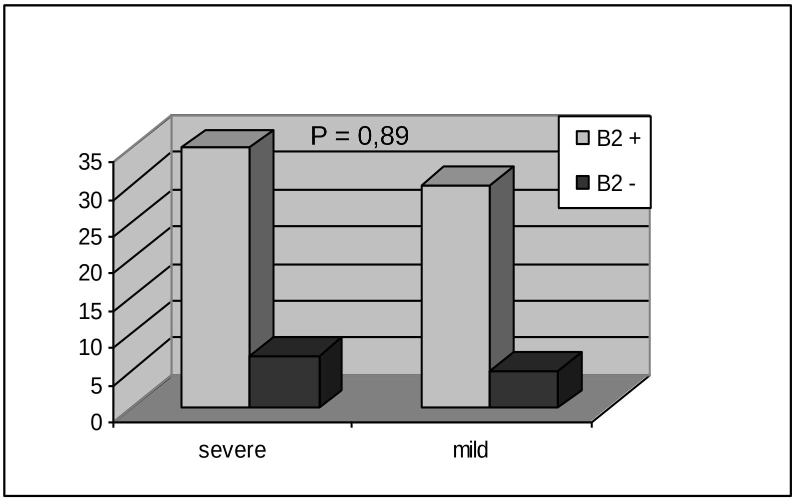

B

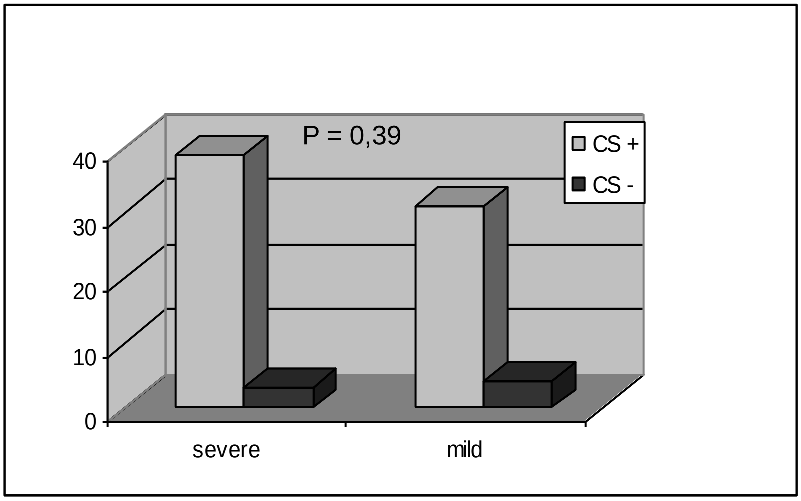

C

Use of drugs in dependence to clinical severity score (RDAI).
No significant association is evident among RDAI score and either antibiotics (A), or bronchodilators (B) or steroids (C). Severe forms are considered forms with RDAI score ≥ 9; mild forms are considered forms with RDAI score ≤8. ATB, antibiotics; B2, bronchodilators; CS, steroids.
