# Supplementary material for: Therapeutic approach to bronchiolitis: why pediatricians continue to overprescribe drugs?
Source: Ital J Pediatr. 2010 Oct 1;36:67. doi: 10.1186/1824-7288-36-67 (PMC2958958; doi:10.1186/1824-7288-36-67)
Supplement: Additional file 3 — Drugs vs. social risk. No statistically significant difference is evident between social risk and use of antibiotics (A), bronchodilators (B), steroids (C). SR: social risk. + = presence, - = absence. [file 1824-7288-36-67-S3.PDF]

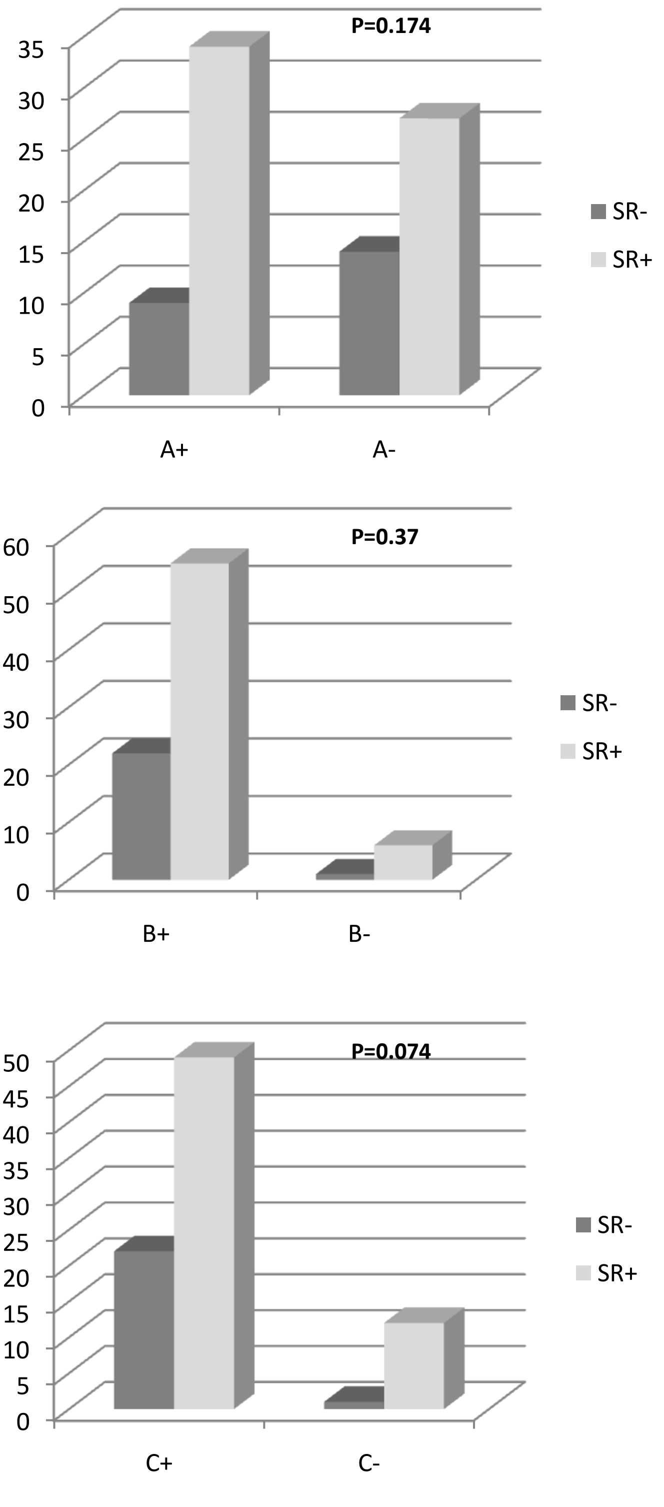

Drugs vs. social risk.

No statistically significant difference is evident between social risk and use of antibiotics (A), bronchodilators (B), steroids (C). SR: social risk. + = presence, - = absence.
